# Hepatic TLR4 signaling is activated by LPS from digestive tract during SARA, and epigenetic mechanisms contribute to enforced TLR4 expression

**DOI:** 10.18632/oncotarget.6161

**Published:** 2015-10-19

**Authors:** Guangjun Chang, Su Zhuang, Hans-Martin Seyfert, Kai Zhang, Tianle Xu, Di Jin, Junfei Guo, Xiangzhen Shen

**Affiliations:** ^1^ College of Veterinary Medicine, Nanjing Agricultural University, Nanjing, China; ^2^ College of Animal Science and Technology, Nanjing Agricultural University, Nanjing, China; ^3^ Leibniz Institute for Farm Animal Biology, Dummerstorf, Germany

**Keywords:** subacute ruminal acidosis, LPS translocation, liver, TLR4 signaling, epigenetic mechanisms, Immunology and Microbiology Section, Immune response, Immunity

## Abstract

Subacute ruminal acidosis (SARA) is known to trigger a systemic inflammatory response that is possibly caused by the translocationof lipopolysaccharides (LPS) from the gastrointestinal tract into the bloodstream. The aim of this study is to investigate this causal relationship between the increases of circulating LPS and liver inflammation. Here we found that SARA goats exhibited significantly increased LPS concentrations in both the rumen and portal vein. The livers of these goats exhibited increased mRNA concentrations of pro-inflammatory genes that indicated inflammation. Meanwhile, the occurrence of liver inflammation was further validated by the enhanced protein expression of those cytokines in the livers of SARA goats. These increased expressions of detected pro-inflammatory genes were likely mediated by enforced TLR4 signaling because SARA increased the concentrations of TLR4 mRNA and protein in the liver and the abundance of both the NF-kB-p65 factor and its active phosphorylated variant. We also verified that the enhanced TLR4 expression was accompanied by chromatin decompaction and demethylation of the proximal TLR4 promoter. Hence, epigenetic mechanisms are involved in the enforced expression of immune genes during SARA, and these findings open innovative routes for interventions via the modulation of these epigenetic mechanisms.

## INTRODUCTION

Subacute ruminal acidosis (SARA) has become the primary metabolic disease in dairy industry and has thus received increasing attention over the last decade [1–3]. The persistent consumption of a high-concentrate (HC) diet by ruminants increases the production of organic acids and causes a depression of the pH in the digestive tract [2–4]. This decrease in pH may result in alterations of the type of fermentation and the composition of the microbiota in the rumen. Together, these changes can eventually lead to the accumulation of lipopolysaccharides (LPS), and may concomitantly disrupt the gastrointestinal barrier and facilitate the translocation of LPS from the digestive tract into bloodstream [5–7]. It is well known that HC-induced SARA increases the plasma concentrations of acute phase proteins (APPs), such as serum amyloid A (SAA), haptoglobin (Hp) and LPS-binding protein (LBP), and elevates the levels of pro-inflammatory cytokines, including interleukin (IL)-1, IL-6 and TNF-α, in the peripheral blood [3, 8, 9]. Hence, the translocation of LPS from the digestive tract into the bloodstream often triggers a systemic inflammatory response [10–12].

LPS-infusion studies have demonstrated that the liver is a prominent site of the synthesis of the pro-inflammatory cytokines that orchestrate the synthesis of hepatic APPs [13, 14]. Toll-like receptors (TLRs) are evolutionarily conserved from the worm to mammals and are responsible for sensing invading pathogens both outside the cell and in the intracellular endosomes and lysosomes [15, 16]. TLR4 is one of 13 different, well-characterized mammalian toll-like-receptors (TLRs), and its signal transduction pathway is known to initiate the activation of nuclear factor (NF)-κB [17]. A large number of inflammation-regulated genes feature NF-κB attachment sites in their proximal promoter regions, and this factor complex functions as a key switch that orchestrates the whole pool of immune response genes [18]. These immune response genes include the cytokine encoding genes whose expressions are strongly increased in the livers of cows as a consequence of LPS-induced mastitis [19, 20]. However, it is unknown whether circulating LPS derived from the digestive tract can enhance the expressions of hepatic immune response genes *via* the TLR4-NF-κB signaling pathway.

Previous studies have demonstrated that the local chromatin structures of promoters and their recruitment of transcriptional factors (TFs), such as NF-κB, are of pivotal importance for regulating gene transcription [21, 22]. The participation of the epigenetic mechanisms of histone modification and DNA methylation in the generation of ‘opened’ and ‘closed’ configurations of chromatin are well documented [22]. Locally open promoter chromatin structures permit the binding of TFs to initiate the transcription of the respective target genes. Previous *in vitro* studies have revealed alterations in the chromatin structure of the TLR4 promoter during LPS infusion-induced LPS tolerance in murine macrophages and human monocytes [23, 24]. However, it is unknown whether LPS derived from the digestive tract during SARA is able to modify the chromatin structure of the TLR4 promoter *in vivo*. Therefore, we hypothesized that the increased translocation of LPS from the digestive tract into liver *via* the portal vein might result in the epigenetically modulated expression of TLR4 and thereby activate the TLR4-NF-κB pathway and ultimately trigger the enhanced expression of immune response genes in this organ.

## RESULTS

### Alterations in rumen pHs, milk yields and milk compositions of goats from control and treatment groups

**Figure 1:**
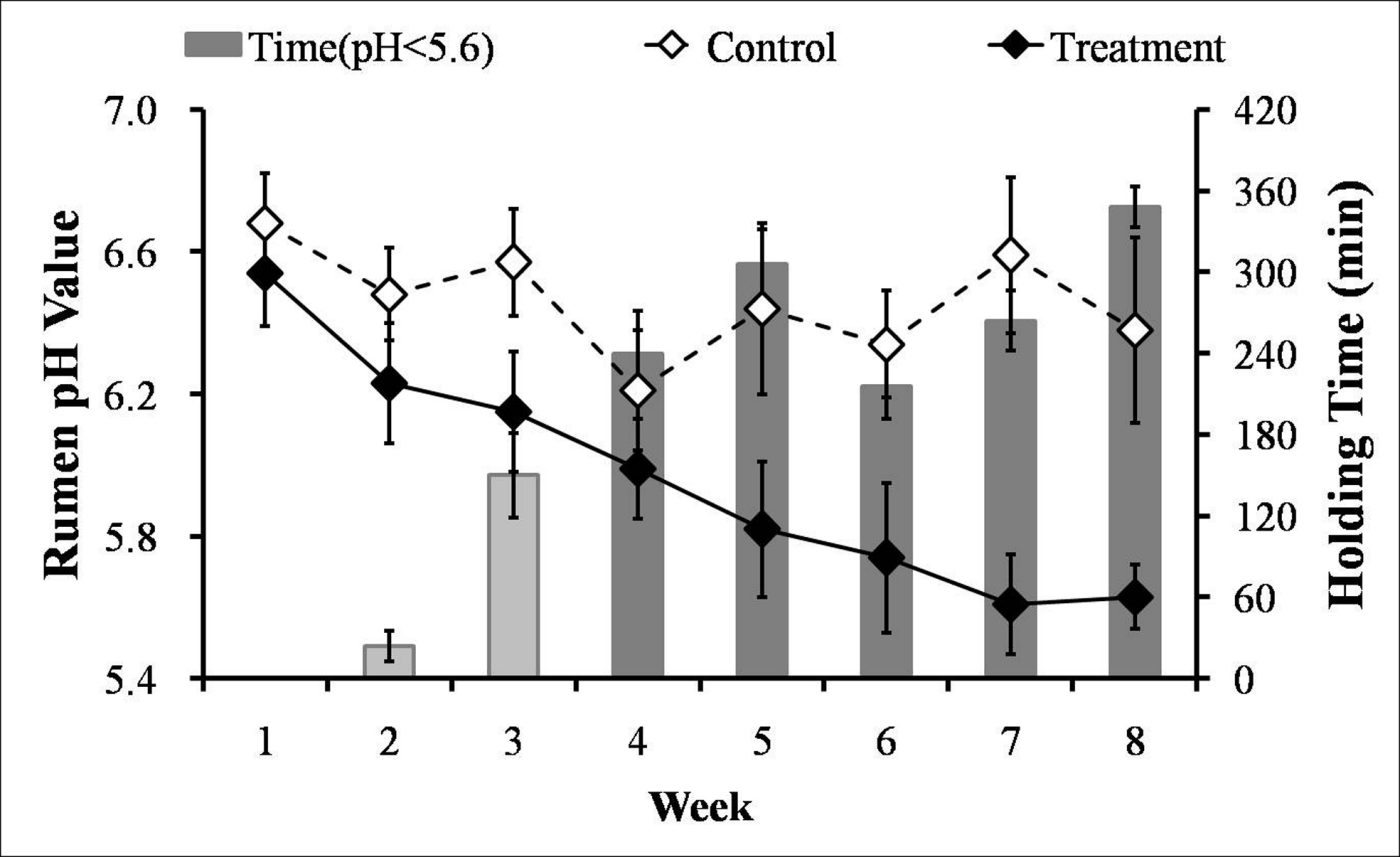
Weekly averages for the rumen pHs and times spent below pH 5.6 for the lactating goats from the control and treatment groups White filled rhomboids, control group (*n* = 6, mean ± SEM); black filled rhomboids, treatment group (*n* = 6, mean ± SEM). Light gray bar, less than 180 min/day with a rumen pH below 5.6; dark gray bar, more than 180 min/day with a rumen pH below 5.6.

The consumption of the HC diet caused a gradual decline in the average daily rumen pH in the treatment group from 6.54 in the 1^st^ week to 5.63 in the 8^th^ week, whereas the pH remained stable and above 6.2 in the control group beginning in the 1^st^ week (Figure 1). From the 4^th^ week onward, the treatment group experienced SARA, as demonstrated by durations of reduced rumen pH values below 5.6 that persisted for more than 180 min/d (Figure 1).

Eight weeks of feeding with the HC diet significantly decreased the average daily milk yield (*P* < 0.01), milk fat percentage (*P* = 0.04) and milk lactose percentage (*P* < 0.01) compared with the LC diet but did not affect the milk protein percentage (*P* = 0.12; Table 2).

**Table 1:** Chemical compositions and nutrient levels of the diets

Ingredient	Percentage (%) of the ingredients in the different diets (based on dry matter)	
	Control diet	Treatment diet
Chinese wildrye hay	40.00	26.70
Alfalfa hay	20.00	13.30
Corn	22.99	23.24
Bran	0	20.77
Soybean meal	15.00	13.66
Limestone	0.65	1.43
Calcium phosphate dibasic	0.46	0.00
Salt	0.4	0.40
Premix ^a^	0.5	0.50
Forage: Concentrate(F:C)	6:4	4:6
**Nutrient level, % of dry matter**		
Net energy, MJ/kg	5.73	5.83
DCP, %	9.90	10.00
NDF, %	36.64	34.55
ADF, %	24.74	20.35
NFC, %	31.76	35.00
Ca, %	0.80	0.90
P,%	0.33	0.38

a The premix provided the following per kg of diet: 3000, 1250, and 40 IU vitamins A, D and E, respectively; and 6.25, 62.5, 62.5, 50, 0.25, 0.125, and 0.125 mg Cu, Fe, Zn, Mn, I, Se, and Co, respectively.

**Table 2:** Milk yields and milk components of the goats from the control and treatment groups

Item	Control ^a^ (*n* = 6)	Treatment ^a^ (*n* = 6)	*P*-value		
			Diet	Week	Diet × Week
Milk Yield (kg/d)	1.33 ± 0.07	1.13 ± 0.06	< 0.01	0.28	0.50
Milk Protein (%)	4.11 ± 0.10	3.88 ± 0.05	0.12	0.09	0.11
Milk Fat (%)	3.36 ± 0.15	2.93 ± 0.11	0.04	0.07	0.97
Milk Lactose (%)	3.31 ± 0.09	3.05 ± 0.05	< 0.01	0.21	0.34

a Mean ± SEM

### LPS concentrations in rumens and portal veins and the concentrations of primary pro-inflammatory cytokines in portal veins of goats in control and treatment groups

The goats fed the HC diet in treatment group exhibited notably higher free LPS concentrations in the rumen compared with the goats fed the low-concentrate (LC) diet in control group (*P* = 0.02). The peak free LPS concentration in the rumen was observed 4 h after feeding (Table 3). The LPS concentrations measured in the portal vein were significantly increased in the treatment group goats compared with the control goats (*P* < 0.01), and on the sampling days, the concentrations in the portal vein exhibited a trend toward an increase with the sampling time (*P* = 0.07; Table 3).

**Table 3:** LPS concentrations in the rumens and portal veins of the goats from the control and treatment groups

LPS Conc.	Control ^a^(*n* = 6)	Treatment ^a^(*n* = 6)	Effect, *P*-value		
			Diet	Time	Diet × Time
Rumen (EU/mL) ^$^					
0 h	3.09 ± 0.57	5.51 ± 1.02	0.02	0.48	0.96
4 h	3.85 ± 0.65	6.41 ± 1.29			
8 h	3.48 ± 0.43	6.27 ± 0.93			
Portal Vein (EU/mL)					
0 h	0.56 ± 0.12	1.02 ± 0.35	< 0.01	0.07	0.69
4 h	0.76 ± 0.11	1.20 ± 0.09			
8 h	0.64 ± 0.06	1.21 ± 0.25			

a Mean ± SEM

$ [× 10^4^]

The plasma concentrations of primary pro-inflammatory cytokines IL-1β (*P* < 0.01), IL-6 (*P* = 0.05) and TNFα (*P* < 0.01) in the portal vein were significantly increased in the treatment group compared to the control group (Table 4).

**Table 4:** The concentrations of primary pro-inflammatory cytokines in the plasma of the portal vein of the goats from the control and treatment groups

Item Conc.	Control ^a^(*n* = 6)	Treatment ^a^(*n* = 6)	Effect, *P*-value		
			Diet	Time	Diet × Time
IL-1β (ng/mL)					
0 h	0.28 ± 0.06	1.56 ± 0.15	< 0.01	0.08	0.11
4 h	0.22 ± 0.03	2.12 ± 0.30			
8 h	0.32 ± 0.06	1.94 ± 0.32			
IL-6 (pg/mL)					
0 h	67.95 ± 13.56	199.89 ± 31.17	0.05	0.06	0.17
4 h	71.01 ± 15.05	238.60 ± 65.24			
8 h	74.73 ± 15.99	235.23 ± 33.18			
TNFα （fmol/mL）					
0 h	15.69 ± 0.67	148.34 ± 27.8	< 0.01	0.10	0.13
4 h	17.09 ± 1.17	167.19 ± 20.33			
8 h	16.48 ± 1.05	125.47 ± 28.52			

a Mean ± SEM

### Expression of detected innate immune genes in the livers of goats in control and treatment groups

**Figure 2:**
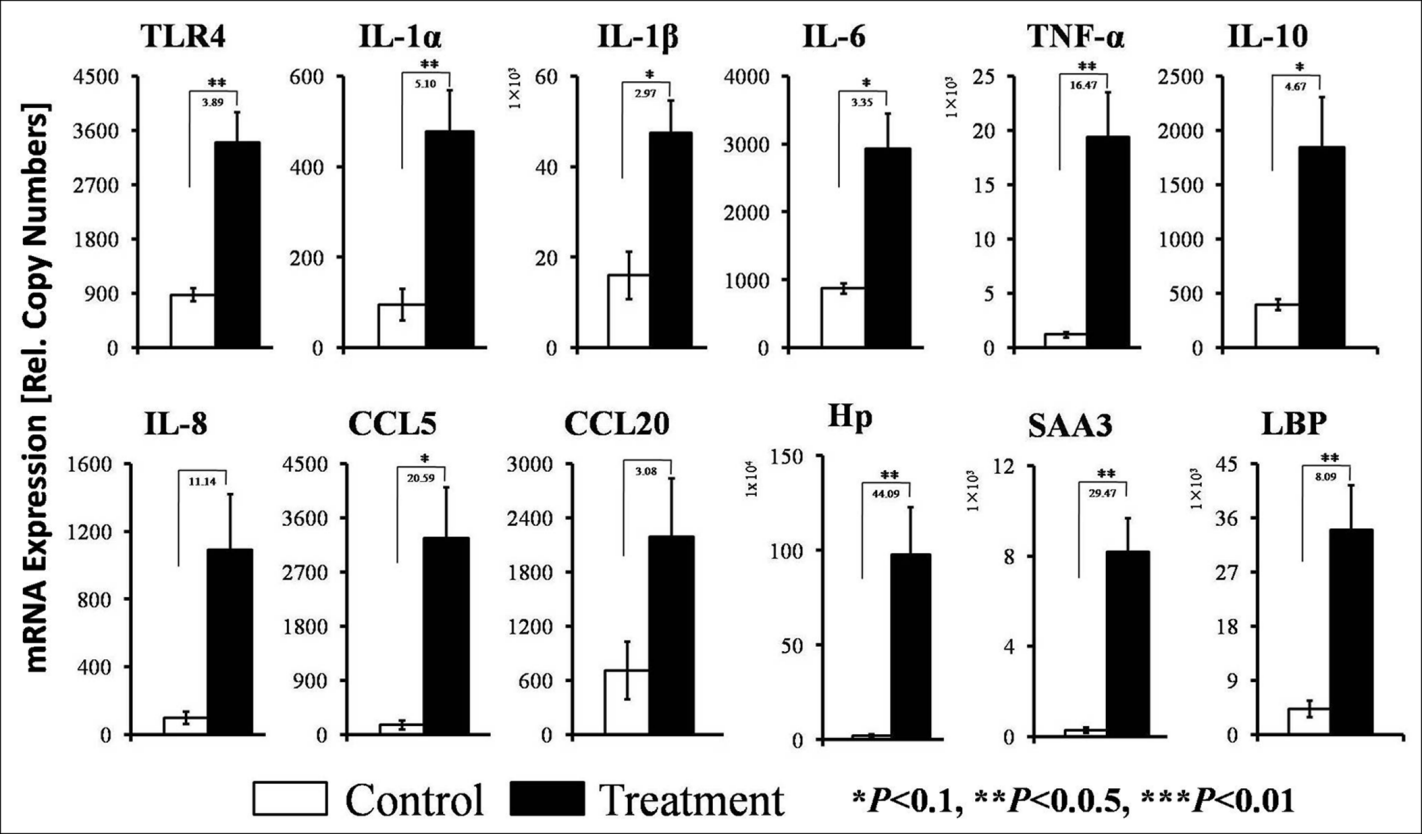
Quantifications of selected innate immune genes by RT-qPCR in the livers of the goats from the control and treatment groups The ordinate axis indicates the relative copy numbers (*n* = 6 in each group, mean ± SEM). White filled bar, control group; black filled bar, treatment group. The fold changes and significance (*P < 0.10, **P < 0.05, ***P < 0.01) of the selected genes between the control and treatment groups are indicated.

The concentrations of mRNAs encoding innate immune genes (i.e., cytokines, chemokines and acute phase proteins) were increased in the treatment goats compared with the control goats (Figure 2). SARA significantly increased the expressions of the pro-inflammatory cytokines IL-1α and TNF-α. The concentrations of IL-1β and IL-6 exhibited increases in the livers of the treatment goats compared with the control goats, and the magnitudes of the increases in pro-inflammatory cytokine expressions differed between the control and treatment groups, as indicated in Figure 2. The expression level of the anti-inflammatory cytokines IL-10 was increased by 5-fold in the treatment group compared with the control group. The expression levels of the key chemokines IL-8, CCL5 and CCL20 were increased by 11-, 21- and 3-fold, respectively, in the treatment group. The expression levels of the acute phase proteins Hp, SAA3 and LBP were significantly elevated by 44-, 29-, 8-fold, respectively, in treatment goats compared with the control goats. The expression of TLR4, which is the specific receptor for LPS, was significantly enhanced by 4-fold in the treatment group compared with the control group.

### TLR4, NF-κB p65 and primary pro-inflammatory cytokines protein expressions in livers of goats from control and treatment groups

**Figure 3:**
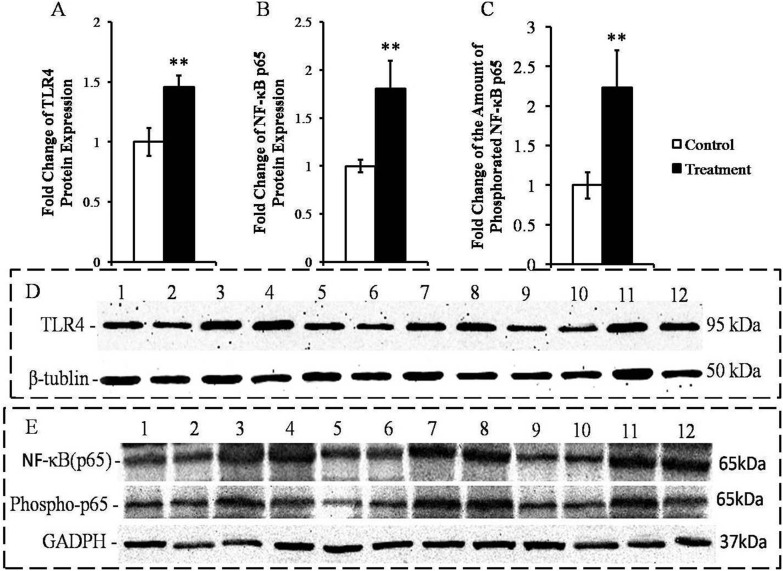
Measurement of the expressions of the TLR4 and NF-κB p65 proteins and the amounts of phospho-p65 protein in the livers of the goats from the control and treatment groups The protein levels are expressed as arbitrary units relative to β-tubulin or GADPH; fold changes in TLR4 **A.**, NF-κB p65 **B.** and phospho-p65 **C.**. D. Western blotting results for TLR4 and β-tubulin. Control group (bands 1, 2, 5, 6, 9, 10); treatment group (bands 3, 4, 7, 8, 11, 12). E. Western blotting results for NF-κB p65, phospho-p65 and GADPH. Control group (bands 1, 2, 5, 6, 9, 10) and treatment group (bands 3, 4, 7, 8, 11, 12). The significance of the changes in protein levels are indicated (*P < 0.10, **P < 0.05 and ***P < 0.01).

HC diet-induced SARA increased the hepatic concentrations of the TLR4 and NF-κB p65 proteins in the treatment goats compared with the control goats (*P* < 0.05; Figure 3A and 3B). Importantly, the amount of phosphorylated NF-κB p65 was higher in the livers of the SARA group animals than in the control animals (*P* < 0.05; Figure 3C).

**Figure 4:**
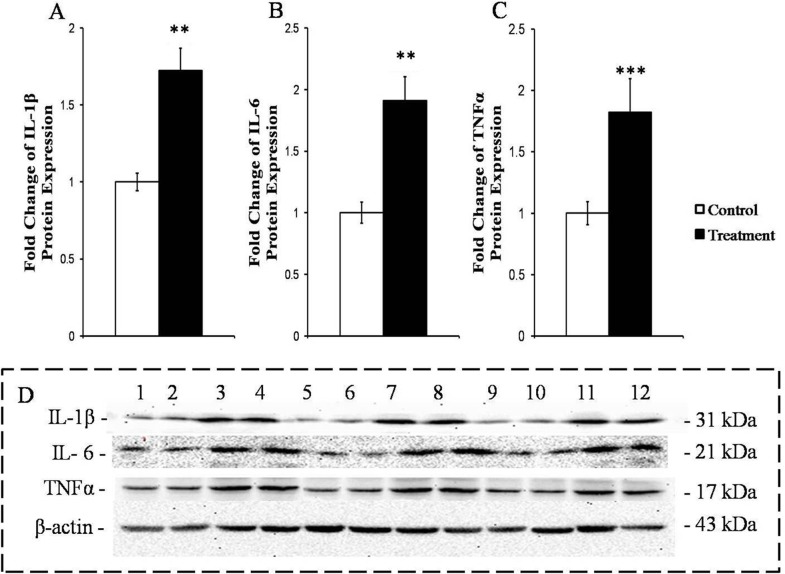
Measurement of the protein expressions of the primary pro-inflammatory cytokines in the livers of the goats from the control and treatment groups The protein levels are expressed as arbitrary units relative to β-actin; fold changes in IL-1β **A.**, IL-6 **B.** and TNFα **C.**. D. Western blotting results for IL-1β, IL-6, TNFα and β-actin. Control group (bands 1, 2, 5, 6, 9, 10); treatment group (bands 3, 4, 7, 8, 11, 12). The significance of the changes in protein levels are indicated (*P < 0.10, **P < 0.05 and ***P < 0.01).

The protein expressions of primary pro-inflammatory cytokines IL-1β (*P* < 0.0.1), IL-6 (*P* < 0.05) and TNFα (*P* < 0.01) were significantly enhanced in the livers of the SARA goats compared to those in the liver of the control goats (Figure 4).

### Chromatin remodeling and DNA methylation in TLR4 promoter region in livers of goats in control and treatment groups

**Figure 5:**
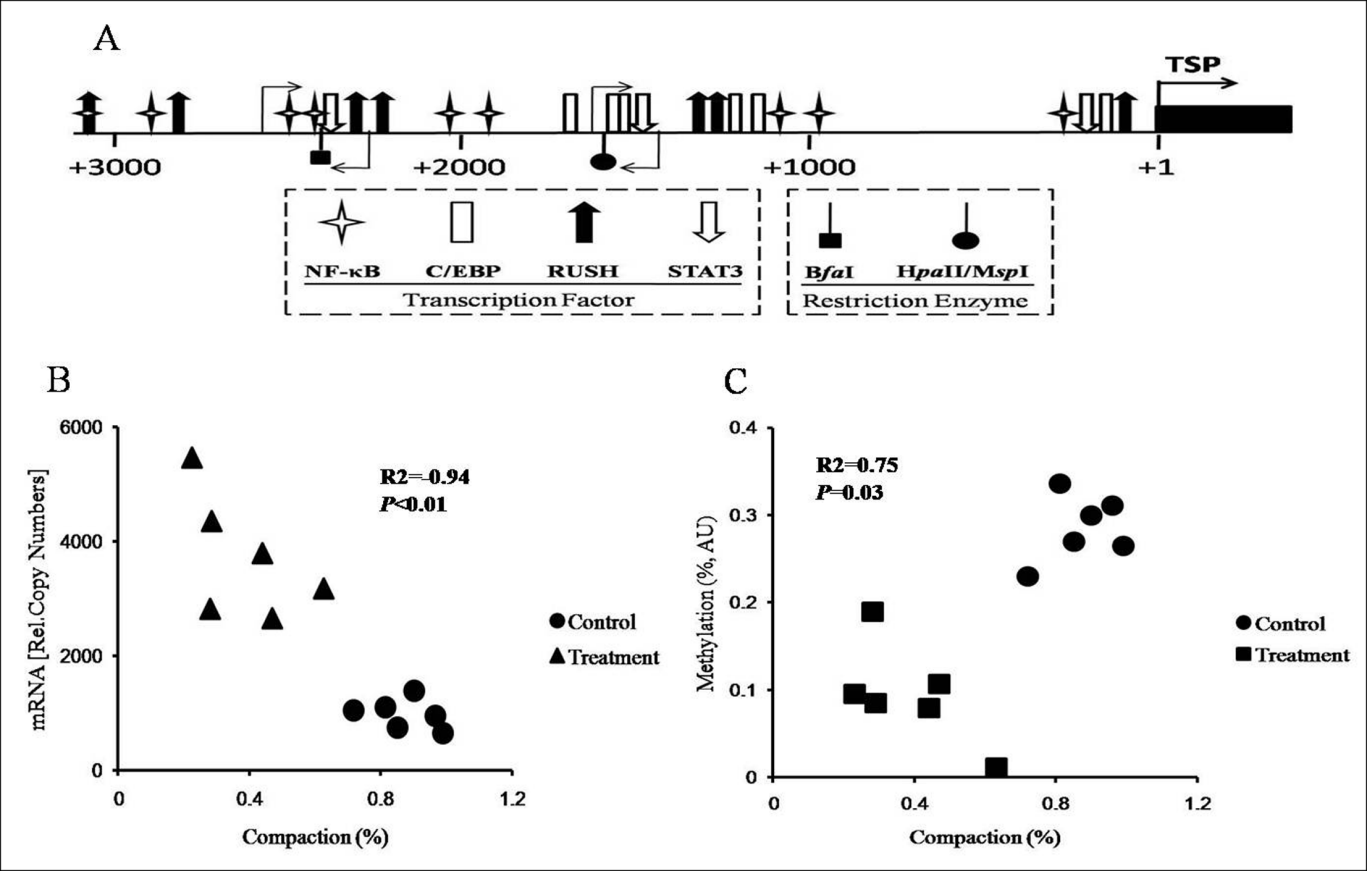
Analysis of chromatin remodeling and DNA methylation in the promoter of TLR4 in the livers of the goats from the control and treatment groups **A.** Map of the positions of the binding sites for the relevant transcription factors in the promoter of TLR4. The numbers refer to the transcriptional start sites (TSP, +1), which are indicated with filled arrows, and the 5′-position of exon 1 is indicated by the black box. The positions of the various transcription factors and restriction enzyme recognition sites are indicated by the respective symbols. The light arrows denote the positions of the primers used for CHART-PCR and the methylation assay. **B.** Correlations between chromatin compaction and mRNA copy numbers. R^2^, coefficient of correlation; *P*, significance of the correlation. **C.** Correlation between chromatin compaction and DNA methylation. R^2^, coefficient of correlation; *P*, significance of the correlation. AU, arbitrary units.

The promoter of TLR4 has not been thoroughly examined in goats. Therefore, we identified the DNA sequence of the TLR4 promoter *via* BLAST analyses using the TLR4 mRNA sequences from the NCBI file NW_005100742.1. Next, we analyzed the potential binding sites for the relevant transcription factors in the TLR4 promoter area *in silico* to verify the core promoter sequence for further analyses of chromatin remodeling and DNA methylation (Figure 5A).

The results of the chromatin accessibility by real-time PCR assay (CHART-PCR) revealed that the average degree of chromatin compaction in the treatment group was significantly lower than that in the control group (*P* < 0.01; Table 5). Plotting the individual degree of chromatin compaction against the mRNA copy number revealed a significant correlation between these TLR4-encoding gene-related parameters (R^2^ = −0.94, *P* < 0.01; Figure 5B). Additionally, the DNA methylation results revealed that the average degree of TLR4 promoter methylation in the treatment group goats was notably decreased compared with that in the control goats (*P* < 0.0.1; Table 5). Plotting the individual degree of chromatin compaction against the degree of DNA methylation revealed a significant correlation (R^2^ = 0.75, *P* = 0.03; Figure 5C).

**Table 5:** The average degrees of chromatin compaction and DNA methylation in the TLR4 promoter region in the livers of the goats from the control and treatment groups

Item	Control ^a^ (*n* = 6)	Treatment ^a^ (*n* = 6)	*P*-value
Chromatin compaction	0.87 ± 0.06	0.40 ± 0.09	< 0.01
DNA methylation	0.29 ± 0.02	0.08 ± 0.02	< 0.01

a Mean ± SEM

## DISCUSSION

SARA is a consequence of rumen fermentation disorder, and it causes a diverse of secondary diseases and enormous economic losses [11]. Rumen pH value is the most widely used parameter to define SARA. In the present study and consistent with the previous reports [10, 25], the average rumen pH was below 5.6 for at least 180 min/d from the 4^th^ week onward in the treatment group, which indicated that SARA was successfully induced. Additionally, in accordance with previous research [3, 10, 26], we observed that experimentally-induced SARA reduced the milk yield and milk fat percentage in the goats fed the HC diet compared with the goats fed the LC diet, although the energy and protein levels were similar between the groups. The reduction in the milk production performance exhibited by the goats fed the HC diet might resulted from the effects of alterations in the type of rumen fermentation type that were caused by the increased concentrate proportion [11].

The induction of SARA *via* feeding with a HC diet is well known to increase the concentration of LPS in the gastrointestinal tract [2, 27], which may disrupt the barrier function of the gastrointestinal epithelium [6, 7, 28, 29] and facilitate the translocation of LPS from the digestive tract into the circulation. The presence of LPS in the blood of lactating goats and cows fed HC diets can increase the concentrations of primary pro-inflammatory cytokines [3, 9]. Our results revealed that the LPS concentrations in the rumen and the portal vein, as well as the concentrations of primary pro-inflammatory cytokines in the plasma of portal vein were significantly increased in the treatment group compared with the control group, which indicated that HC-induced SARA led to the translocation of LPS from the digestive tract into the portal vein and thereby increased the entry of LPS into the liver to enhance the secretion of pro-inflammatory cytokines.

Our RT-qPCR-based determination of the markers of inflammation in the liver clearly verified that the animals suffered from liver inflammation. This finding accord with previous findings from lactating goats suffering from HC-diet induced SARA [3]; these goats also displayed significantly enhancement in the expressions of several genes encoding pro-inflammatory cytokines and acute phase proteins (APPs). We identified four lines of evidence that suggest that TLR4 signaling was crucially involved in the induction of this inflammatory response in the liver. First, lipoprotein A from LPS is known to be the only ligand of and highly specific for the TLR4 receptor [16, 30], and we observed increased concentrations of LPS in the portal veins of the treatment group animals. Second, we found that SARA increased the mRNA and protein concentrations of TLR4 in liver. Third, LPS-triggered TLR4 signaling is known to ultimately activate the NF-κB factor complex [31, 32], and we observed that the abundances of both NF-κB (p65) and phosphorylation-activated phosphor-p65 were increased in the livers of the treatment group goats. Fourth, the activation of the NF-κB transcription factor complex is known to be a key determinant of immune defense because these factors collectively control the expression of more than 100 immune genes [18, 33], and our results clearly showed that the expressions of pro-inflammatory cytokines mRNA and protein were significantly increased in the livers of the SARA goats compared to the control goats. Together, these diverse lines of evidence strongly suggest that the LPS-mediated induction of TLR4 signaling caused the inflammation of the livers of the goats suffering from SARA.

We examined the degrees of chromatin compaction in the relevant areas of the TLR4 promoter to determine the contribution of chromatin remodeling to the regulation of the expression of immune genes during SARA. Our combined observations of (i) chromatin decompaction of the target area and (ii) decreased CpG methylation clearly verified that epigenetic mechanisms of gene expression control were triggered in the group of goats suffering from diet-induced SARA. This results of the current study conform well with our previous finding that the systemic reaction that occurs during mastitis that is experimentally induced with *E. coli* in dairy cows triggers chromatin decompaction at the TLR4 promoter in the liver. This decompaction is accompanied by DNA demethylation and enhanced TLR4 expression [34]. The common factor in both of our studies may have been the systemically increased levels of LPS circulating in the blood. These findings suggest that systemic increases in LPS, regardless of whether they are due to the liberation of LPS during SARA or the conceivable liberation of LPS during mastitis, have widespread detrimental effects on the proper functioning of the liver. The LPS-mediated effects trigger epigenetic mechanisms in the liver.

In conclusion, our study clearly documented that an important detrimental feature of diet-induced SARA is to causing liver inflammation. This inflammation is triggered by increased levels of circulating LPS that, in turn, conceivably activate excessive TLR4 signaling, and thereby enhance the expressions of innate immune genes and increase the secretions of pro-inflammatory cytoines in this central metabolic organ. The widespread consequences of liver dysfunction may also influence milk production. Our study also indicates that epigenetic mechanisms are involved in the enfored expression of innate immune genes, and these findings open innovative routes for feasible interventions based on the applications modulators of epigenetic mechanisms, such as histone deacetylases and demethylases and their inhibitors [35, 36].

## MATERIALS AND METHODS

### Animals, diets, and experimental design

The goats were housed in individual metabolic cages in the Centre of Experimental Animals at Nanjing Agricultural University (Nanjing, China) in accordance with the guidelines of the Animal Ethics Committee at Nanjing Agricultural University and in compliance with the Regulations for the Administration of Affairs Concerning Experimental Animals (the State Science and Technology Commission of People's Republic of China, 1988). All experimental protocols were approved by the Animal Care and Use Committee of Nanjing Agricultural University, 1999. Twelve multiparous lactating goats (3-5 weeks post-partum, aged 2-3 years) exhibited an averaged milk yield of 2.16 ± 0.96 kg/day and average body weights of 42.32 ± 2.8 kg at the beginning and 40.28 ± 4.18 kg at the end of the experiment. The goats were randomly assigned to either a treatment group that was fed a HC diet (forage:concentrate = 40: 60) to induce SARA or to a control group that was fed a LC diet (forage:concentrate = 60:40) (Table 1). Prior to the initiation of the experiment, all of the goats were ruminally cannulated, fitted with indwelling catheters in the portal vein, and fed an LC diet for a 4-week adaption phase to ensure the similarity of the rumen fermentation statuses. The catheters were serviced by the infusion of sterilized heparin saline (250 IU/mL) three times daily at 8-h intervals. After the surgery and an adaption phase, the goats exhibited an average milk yield of 1.63 ± 0.58 kg/day and received the appropriate diet for 8 weeks. The goats were fed at 8:30 and 16:30 daily and were provided free access to fresh water for the duration of the experiment. Additionally, body temperatures and milk SCCs were monitored weekly udder health parameters because mastitis can affect the LPS concentration in the blood and other experimental parameters. Diet and dietary ingredient samples were collected weekly and analyzed by wet chemistry as previously described [37].

### Rumen pH and milk measurements

The rumen pH values were measured on the last 2 consecutive days of each week during the experiment. On these days, rumen fluid was collected at 30-min intervals for 24 h. The pH data were summarized first as the average pHs and second as the times spent below pH 5.6 for each week. The goats were milked twice daily at 8:30 and 16:30, and the milk yields were recorded. Milk samples (50 mL) were collected from the last three consecutive days of each experimental week. These samples were preserved with potassium dichromate and stored at 4°C until analysis with a FossMatic 5000 analyzer (FOSS Electric A/S, Denmark) to determine the milk fat, protein and lactose contents.

### Rumen fluid, blood and liver tissue sampling

Rumen fluid and portal vein blood samples were collected from all goats 15 min prior to feeding (0 h) and at 4 h and 8 h after feeding on the last 3 consecutive days of the 8^th^ week. The protocols for the preparing rumen fluid and blood samples for laboratory analyses have been described previously [38].

After sampling of the rumen fluid and blood, the goats were slaughtered, and the liver tissue was then immediately excised. Small cubes (2 mL) were snap-frozen and stored in liquid nitrogen.

### LPS and primary pro-inflammatory cytokines measurement

The LPS concentration was measured with a chromogenic endpoint assay (cat. CE64406, Chinese Horseshoe Crab Reagent Manufactory Co., Xiamen, China) with minimum detection limits of 0.05 EU/mL (rumen liquid) or 0.01 EU/mL (plasma). The procedures were performed according to the manufacturer's instructions as described by Chang *et al*. [39].

The concentrations of primary pro-inflammatory cytokines IL-1β, IL-6 and TNF-α in the plasma of portal vein were measured by Radioimmunoassay with commercially available human radioimmunoassay kits purchased from Beijing North Institute of Biological Technology. The detection range of radioimmunoassay kits for IL-1β (cat. C09DJB), IL-6 (cat. C12DJB) and TNF-α (cat. C06PJB) were 0.1-8.1 ng/mL, 50-4000 pg/mL and 9-590 fmol/mL, respectively. This method has been described by Chang *et al* [39].

### RNA extraction and RT-qPCR

RT-qPCR was performed using an ABI 7300 system (Applied Biosystems, Foster City, CA, USA) to determine the relative copy numbers of the different mRNA transcripts. The liver samples were powdered in small tubes (5 mL), and the total RNA was extracted using TRIZOL (Takara, Dalian, China) in accordance with the manufacturer's protocol. For the cDNA synthesis, 1.5 μg total RNA was prepared in a reverse transcription reaction (cat. RR036A, Takara) with oligo-dT for all mRNAs. RT-qPCR was performed using gene-specific primer pairs to amplify the cDNA target segments with SYBR Premix EX Taq™ kit (cat. DRR420A, Takara). The relative copy numbers of the individual mRNA were calculated against the dilution series (10^6^ to 10^2^ copies) of the respective cloned amplicons as external standards. The amplification primers are listed in the Supplementary Table.

### Chromatin preparation and CHART-PCR

A 100-mg tissue sample was powdered in liquid nitrogen and suspended in 3 mL pre-chilled resuspension buffer (RSB; 10 mM Tris [pH 8.0], 3-mM MgCl_2_, and 10-mM NaCl) containing 0.5% Nonidet NP 40. A freshly prepared dilution (1/200) of a proteinase inhibitor cocktail (REF: 04693132001, Roche) and phenyl-methyl-sulfonyl-fluoride (PMSF; 1 mM)) was added immediately prior to use. After incubation for 5 min on ice, the tissue powder was homogenized using a Dounce homogenizer (Sigma, D9063). The liquid was filtered through sterilized glass wool into a pre-cooled 15 mL Eppendorf tube. Next, the filtrate was centrifuged at 1,000 *×g* for 10 min at 4°C to pellet the nuclei. The pellets were washed once in RSB buffer containing 1 mM β-mercaptoethanol and subsequently transferred into a pre-cooled 1.5-mL Eppendorf vial. The nuclei were pelleted again (1,000 *×g*, 5 min, 4°C) and re-suspended in 100 μL RSB buffer containing 50 % glycerol and stored at −20°C.

Chromatin compaction was measured using the CHART-PCR [40] as described Vanselow *et al*. [41]. The restriction enzyme used to determine the amount of chromatin compaction at the TLR4 promoter was M*ae*I. The quantity of undigested target DNA was measured by real-time PCR (ABI 7300) using a protocol similar to that described above for the RT-qPCR. The primers used to amplify the target promoter sequence of the TLR4 encoding gene (NW_005100742.1) were 5′-CATAACAGCACTTCAAGGTAC (forward) and 5′-GGAAGCTGCTATGCATTAGAT (reverse). The degree of compaction is represented as the fraction of the copy numbers determined from the digested *vs* undigested control samples.

### Methylation assay of the TLR4 promoter

Genomic DNA was extracted from the liver tissues and analyzed with Methyl-Profiler DNA Methylation RT-qPCR Assays with some modifications [42, 43]. This method exploits the fact that H*pa*II digestion is blocked by the methylation of C nucleotides in CpG dinucleotides, whereas its isoschizomer M*sp*I is not blocked. Purified genomic DNA was predigested with E*co*RI to facilitate accurate aliquoting. Three micrograms of the digested and subsequently purified DNA were distributed and digested with H*pa*II or M*sp*I (10 U, 37°C, 2 h). The control was similarly treated, but the enzyme was not added. Subsequently, the DNA was re-purified and re-quantified, and 200 ng (equivalent to ~6,000 gene copies) was used to determine the remaining DNA copies for the qPCR assays. The primers used to amplify the target sequence of the TLR4 promoter were 5′-GCTTTGTCTATGCAGTCACTT (forward) and 5′-CAGTCCCTGCTCCAGGAAGAT (reverse). The degree of methylation was calculated based as the ratio of the copy numbers obtained following H*pa*II and M*sp*I digestions, and each value is presented relative to the value obtained for the undigested control (set as 100 % of the input DNA). These calculations have previously been described in detail [34]

### Western blotting analyses of TLR4, NF-κB (p65), NF-κB (phospho-p65), IL-1β, IL-6 and TNFα

The total proteins were extracted from finely minced liver tissue with RIPA Lysis Buffer (cat. SN338, Sunshine Biotechnology Co., Nanjing, China). The protein concentrations were determined the BCA assay (Pierce, Rockford, IL, USA). A total of 50 μg of protein extract from each sample was boiled in Laemmli sample buffer [44] and loaded onto a 7.5 %, 10 % or 15 % SDS-PAGE gel. The separated proteins were transferred onto nitrocellulose membranes (Bio Trace, Pall Co., Port Washington, NY, USA). After transfer, the membranes were blocked for 2 h at room temperature in blocking buffer (skim milk powder) and subsequently incubated with the following primary antibodies in dilution buffer overnight at 4°C: m-anti-TLR4 (1:200; sc-293072, Santa Cruz), rb-anti-NF-κB p65 (1:500; AN365, Beyotime, China), rb-anti-NF-κB phospho-p65 (ser536; 1:500; AN371, Beyotime, China), rb-anti-IL-1β (1:200; sc-7884, Santa Cruz), rb-anti-IL-6 (1:200; sc-1265, Santa Cruz), rb-anti-TNFα (1:200; sc-8301, Santa Cruz), m-anti-β-Tubulin (1:5,000; KC-5T01, Kang Chen Bio-tech, China), rb-anti-GADPH (1:5,000; AP0066, Bioworld, USA), and rb-anti-β-actin (1:500; sc-130656, Santa Cruz). After washing 6 times in Tris-buffered saline with Tween (TBST), the membranes were incubated with goat anti-rabbit or goat anti-mouse horseradish peroxidase (HRP)-conjugated secondary antibodies (1:10,000; SunshineBio, China) in dilution buffer for 2 h at room temperature. Next, the blots were washed as previously described and developed with enhanced chemiluminescence (ECL) using the LumiGlo substrate (Super Signal West Pico Trial Kit, Pierce). The ECL signals were recorded using an imaging system (Bio-Rad, Hercules, CA, USA) and analyzed using the Quantity One software (Bio-Rad).

### Statistical analysis

The data from milk and blood samples were analyzed as repeated measures using the MIXED procedures of SAS (SAS version 9.2, SAS Institute Inc.). For milk yield, milk fat, milk protein and milk lactose, the effects of diet and week were considered fixed, and the effect of week was analyzed as a repeated measure. The effect of goat was considered a random effect. For the LPS concentrations of the rumen and portal vein, as well as the concentrations of primary pro-inflammatory cytokines in the plasma of the portal vein, the effects of diet and sampling time were considered fixed, and the effect of sampling time was analyzed as a repeated measure. The effect of goat was also considered random. Additionally, the data regarding the mRNA and protein expressions, the degree of chromatin compaction, and the percentage of promoter methylation were analyzed using the ANOVA package of SAS. The correlation coefficient between the mRNA expression levels and the degree of chromatin compaction and between the degree of chromatin compaction and the percentages of promoter methylation were analyzed using Pearson's model in SAS. The effects were considered significant at *P* < 0.05 and highly significant at *P* < 0.01. Trends were discussed at *P* < 0.10.

## SUPPLEMENTARY MATERIAL TABLE


