# Supplementary material for: Hepatic TLR4 signaling is activated by LPS from digestive tract during SARA, and epigenetic mechanisms contribute to enforced TLR4 expression
Source: Oncotarget. 2015 Oct 19;6(36):38578–90. doi: 10.18632/oncotarget.6161 (PMC4770722; doi:10.18632/oncotarget.6161)
Supplement: Supplementary file 1 [file oncotarget-06-38578-s001.pdf]

Hepatic TLR4 signaling is activated by LPS from digestive tract during SARA and epigenetic mechanisms contribute to enforced TLR4 expression

Supplementary Material

Supplementary Table. The list of primers for amplification of RT-qPCR

Gene	Genebank accession	Forward primer	Reverse primer	Length (bp)
TLR4	NM_001285574.1	CTGAGAACCGAGAGCTGGGAC	GCCTTGAAATGTGTTGTCTTCA	207
IL-1α	NM_001009808.1	GATGATGACCTGGAAGCCATTG	GCTGAGAATCCTCTTCTGATAC	259
IL-1β	NM_001009465.2	CCGTGATGATGACCTGAGGAG	CAAGACAGGTATAGATTCTTGTC	303
IL-6	NM_001009392.1	CGAAGCTCTCATTAAGCACATC	CCAGGTATATCTGATACTCCAG	241
TNF-α	X56756.1	CAACAGGCCTCTGGTTCAGAC	GGACCTGCGAGTAGATGAGG	209
IL-8	XM_005681749.1	CTGAGAGTTATTGAGAGTGGGC	CAGTACTCAAGGCACTGAAGTAG	259
IL-10	XM_005690416.1	GTGATGCCACAGGCTGAGAAC	GAAGATGTCAAACTCACTCATGG	213
CCL5	XM_005693201.1	CTACACCAGCAGCAAGTGCT	CAAGCTGCTTAGGACAAGAGG	190
CCL20	XM_005676644.1	GAAGCAGCAAGCAGCTTTGAC	GTTCCATTCCAGGGAGCATC	244
SAA3	EF564270.1	GACATTCCTCAGGGAAGCTG	CTTCGAATCCTTCCGTACCTG	247
Hp	XM_005692202.1	GGAGTACTCGGTTCGCTATCA	CCATCGTTCATTGATGAGTGTG	280
LBP	XM_005688551.1	GAGCTGTCCACCACCAAGATG	CACACTCAGATCAAATGTACCG	243
